# BCI Performance and Brain Metabolism Profile in Severely Brain-Injured Patients Without Response to Command at Bedside

**DOI:** 10.3389/fnins.2018.00370

**Published:** 2018-06-01

**Authors:** Jitka Annen, Séverine Blandiaux, Nicolas Lejeune, Mohamed A. Bahri, Aurore Thibaut, Woosang Cho, Christoph Guger, Camille Chatelle, Steven Laureys

**Affiliations:** ^1^GIGA Consciousness, Coma Science Group, University and University Hospital of Liège, Liège, Belgium; ^2^Disorders of Consciousness Care Unit, Centre Hospitalier Neurologique William Lennox, Université Catholique de Louvain, Ottignies-Louvain-la-Neuve, Belgium; ^3^Institute of Neuroscience, Université Catholique de Louvain, Brussels, Belgium; ^4^GIGA-Cyclotron Research Centre in vivo Imaging, University of Liège, Liège, Belgium; ^5^g.tec Medical Engineering GmbH, Schiedlberg, Austria; ^6^Guger Technologies OG, Graz, Austria; ^7^Laboratory for NeuroImaging of Coma and Consciousness, Department of Neurology, Massachusetts General Hospital, Harvard Medical School, Boston, MA, United States

**Keywords:** covert command following, P3, FDG-PET, disorders of consciousness, consciousness, brain computer interface

## Abstract

Detection and interpretation of signs of “covert command following” in patients with disorders of consciousness (DOC) remains a challenge for clinicians. In this study, we used a tactile P3-based BCI in 12 patients without behavioral command following, attempting to establish “covert command following.” These results were then confronted to cerebral metabolism preservation as measured with glucose PET (FDG-PET). One patient showed “covert command following” (i.e., above-threshold BCI performance) during the active tactile paradigm. This patient also showed a higher cerebral glucose metabolism within the language network (presumably required for command following) when compared with the other patients without “covert command-following” but having a cerebral glucose metabolism indicative of minimally conscious state. Our results suggest that the P3-based BCI might probe “covert command following” in patients without behavioral response to command and therefore could be a valuable addition in the clinical assessment of patients with DOC.

## Introduction

Severely brain-injured patients with disorders of consciousness (DOC) can be distinguished by their ability to show either only reflexive and thus unconscious behavior (unresponsive wakefulness syndrome, UWS) (Laureys et al., 2010), or more purposeful reactions to the environment without (minimally consciousness state minus, MCS–) or with signs of language preservation such as response to command (minimally consciousness state plus, MCS+) (Giacino et al., 2002; Bruno et al., 2012). A clinical challenge presents itself when diagnosing patients correctly, yet, accurate diagnosis is key for treatment and prognosis. Indeed, patients with residual consciousness have increased chances of recovery and respond better to various treatments such as tDCS (Thibaut et al., 2014), possibly modulating cortical excitability in DOC patients (Bai et al., 2017a), and amantadine (Maythaler et al., 2002).

Structured behavioral assessment, such as the Coma Recovery Scale-Revised (CSR-R), led to an important reduction of the misdiagnosis rate (Schnakers et al., 2009), especially when the behavioral assessment is repeated at least five times (Wannez et al., 2018). In addition, passive neuroimaging techniques can quantify structural and functional brain damage, and could ultimately be used as supplemental tools for diagnosis (Rosanova et al., 2012; King et al., 2013; Demertzi et al., 2015; Chennu et al., 2017). Among them, 18F-fluorodeoxyglucose positron emission tomography (FDG-PET) has been used to indicate that the absence of overt signs of consciousness does not necessarily indicate that the patient is unconscious (Stender et al., 2014). Resting state EEG can be used to passively assess DOC patients' consciousness level, for which spectral measures and functional connectivity are most successful and widely employed (for review see Bai et al., 2017b).

Active ways of assessing covert consciousness and command following are more challenging as it necessitates cognitive integrity for command following (e.g., language comprehension, memory) (Andrews et al., 1996). However, it brings additional key information as patients showing early signs of (covert) command following have a better chance of good outcome (Whyte et al., 2001). Furthermore, command following can potentially be used to establish functional communication which could dramatically increase the patient's quality of life.

About one decade ago, the first evidence for “covert command following” in absence of overt command following was reported using functional MRI (Owen et al., 2006), further used a couple of years later to enable an MCS– patient to functionally communicate (Monti et al., 2010; Bardin et al., 2011). However, fMRI is expensive and hardly accessible for repeated assessments. For this reason, other techniques that can measure voluntary responses not observable at bedside have been used to assess “covert command following.” EEG-based detection of motor imagery showed their potential to establish command following in about 20% of the patients with DOC (Cruse et al., 2011, 2012). The P3 event related potential (ERP), which is observed about 300–500 ms after the presentation of a deviant sensory stimulus in a train of standard stimuli, reflects the novelty of the stimulus. The P3 can be present in varying contexts and levels of consciousness, for example in response to the subjects' own name (Perrin et al., 2006; Li et al., 2015), and it is less sensitive than spectral and connectivity measures in discriminating between UWS and MCS patients (Sitt et al., 2014). Nevertheless, it is also known that attention (which requires consciousness, by definition) can modify the amplitude of the P3 (for review Chennu and Bekinschtein, 2012). Other systems, that do not depend on brain activity directly, used subliminal limb movements (i.e., electromyogram; Habbal et al., 2014; Lesenfants et al., 2016), modulation of breathing (Charland-Verville et al., 2014) or of pH saliva (Wilhelm et al., 2006), pupil dilation during mental effort (Stoll et al., 2013) for detecting command following and communication in DOC or locked in syndrome patients (i.e., fully paralyzed but conscious). However, all these techniques are relying on experts for data acquisition and offline data analysis, and tools that can be directly implemented in clinical setting for non-experts are needed.

In this prospective study, we used a commercially available P3-based BCI system with direct feedback about the patient's performance in clinically well-characterized patients with DOC. Our aim was to identify patients with signs of “covert command following,” and compare those results to cerebral glucose metabolism preservation as measured with FDG-PET (Stender et al., 2014). A secondary aim was to investigate whether there is a relationship between the BCI performance and the level of consciousness (as defined by the CRS-R and the FDG-PET) at the group level.

## Methods

### Subjects

The study was conducted from November 2015 till July 2016 and included a convenience sample of 12 adult patients. Inclusion criteria were patients with DOC without response to command (i.e., UWS or MCS–) after a period of coma and the availability of FDG-PET within 1 week of the BCI assessment. Exclusion criteria were being less than 16 years old, history of developmental, neurologic, or major psychiatric disorder resulting in functional disability before the insult, and being in a (sub-)acute stage after injury (<3 months). All patients were hospitalized for 1 week in the University Hospital of Liège for a thorough clinical assessment of their medical and cognitive status. This assessment included FDG-PET, MRI, EEG and repeated behavioral assessments with the CRS-R. Diagnosis of UWS or MCS– was based on the best out of a minimum of five CRS-R assessments during this 1-week hospitalization. The ethics committee of the Faculty of Medicine of the University of Liège approved the study, and written informed consent was obtained from the patient's legal representative in accordance with the Declaration of Helsinki.

### BCI assessment and data processing

Hard- and software were developed by g.tec (mindBEAGLE g.tec Guger Technologies OG, Graz, Austria). Data were recorded from 8 active gel electrodes (Fz, Cz, C3, C4, CPz, CP1, CP2, Pz) sampled at 256 Hz, referenced to the mastoids, and filtered between 0.1 and 30 Hz using a Butterworth 4th order filter. The BCI analyzed the P3 ERP for the assessment of “covert command following” and potentially communication.

The employed oddball paradigms administered mechanical vibrations with a frequency of 225 Hz, which lasted for 30 ms, with an inter-stimulus interval of 270 ms. A total of 480 stimuli were presented, resulting in a paradigm duration of 2.4 min. In the first paradigm, the vibrotactile with two stimuli (VT2), stimuli were presented on the left (probability of 7/8) and right (probability of 1/8) wrist. Before the start of the session, the patient was aroused if needed (i.e., the patient presented multiple episodes of eye closure during the CRS-R before the BCI assessment) and instructed to mentally count the stimuli presented on the right wrist. If the patient showed eye closure lasting longer than 10 s, the paradigm was paused, the patient was aroused (using the CRS-R arousal facilitation protocol) and the instructions were repeated before continuation of the paradigm. In case of a BCI performance above 70% during the VT2 paradigm (without artifacts from the mechanical vibrations), the result was considered above chance level and the test was extended with a third stimulator (VT3). The threshold of 70% was chosen because it is suggested to be the minimal required performance allowing effective communication using a BCI (Noirhomme et al., 2015). The VT3 paradigm includes a stimulator on the right foot which then acts as standard stimulus (probability of 6/8), and the stimulators on the left and right wrists deliver deviant stimulations each with a probability of 1/8. The subject was instructed through headphones which hand to attend for every block, and mentally count the number of deviant stimulations. Four blocks of 15 target deviant (and 15 non-target deviant plus 90 standard) trials randomly assigned to the left and right wrist, were presented. After this initial training phase, 6 autobiographical questions were asked to the patient. In order to answer, the patient was instructed to concentrate on the left hand for answering “yes,” and on the right hand for answering “no” during a 30-s period.

Data for ERP's was extracted from −100 to 600 around stimulus onset. Trials with an amplitude exceeding 100 μV were rejected from the further analysis. Baseline correction was done using the 100 ms before stimulus onset. The 600 ms after stimulus onset was down sampled to 7 samples. The data processing classified deviant trials using a linear discriminant analysis with 56 features (7 time-points of the down-sampled ERP, for 8 channels). The BCI performance (i.e., the percentage of detected deviant trials), ranging from 0 to 100%, was calculated using a 10-fold cross-validation. For more detailed information on the stimulus presentation and analysis, please refer to previous studies (Ortner et al., 2014; Guger et al., 2017).

### FDG-PET acquisition and processing

Resting 18F-FDG-PET acquisition was performed about 30 min after intravenous injection of approximately 150 MBq radioactive labeled glucose (Gemini TF PET-CT scanner, Philips Medical Systems) in order to quantify cerebral glucose uptake. A low dose CT was acquired prior the 12-min emission scan and used for attenuation correction. PET images were reconstructed using the iterative LOR RAMLA algorithm and correction for dead-time, random events and scatter were applied.

Preprocessing and statistical analysis were done in the Statistical Parametric Mapping toolbox (SPM12, www.fil.ion.ucl.ac.uk/spm) implemented in MATLAB (R2017a). Preprocessing was done as described previously (Stender et al., 2014). Briefly, images were manually reoriented according to the SPM12 FDG-PET template, spatially normalized (using a template for patients and controls) and smoothed (with a 14 mm FWHM Gaussian kernel).

### Statistics

We identified regions that showed preserved cerebral glucose metabolism in patients who showed “covert command following” as compared with patients with a FDG-PET typical for MCS (Stender et al., 2014) who did not show signs of “covert command following.” This was done using a factorial design with four design matrices. Clusters with preserved metabolism were considered significant at FWE *p* < 0.05. The mean glucose uptake (in MBq/cc) of the largest significant cluster was extracted for these six subjects using Marsbar (version 0.44, http://marsbar.sourceforge.net/).

Additionally, for every subject, we identified regions with relative preserved metabolism compared to 34 healthy subjects to obtain a FDG-PET-based diagnosis, as described in more details elsewhere (Stender et al., 2014). A Wilcoxon rank-sum test and chi-square test were used to assess the difference in age and gender between patients and healthy subjects (the latter solely used for the FDG-PET analysis). The CRS-R and FDG-PET based diagnosis were confronted to the VT2 BCI performance at the group level using a Wilcoxon rank-sum tests.

## Results

**Figure 1:**
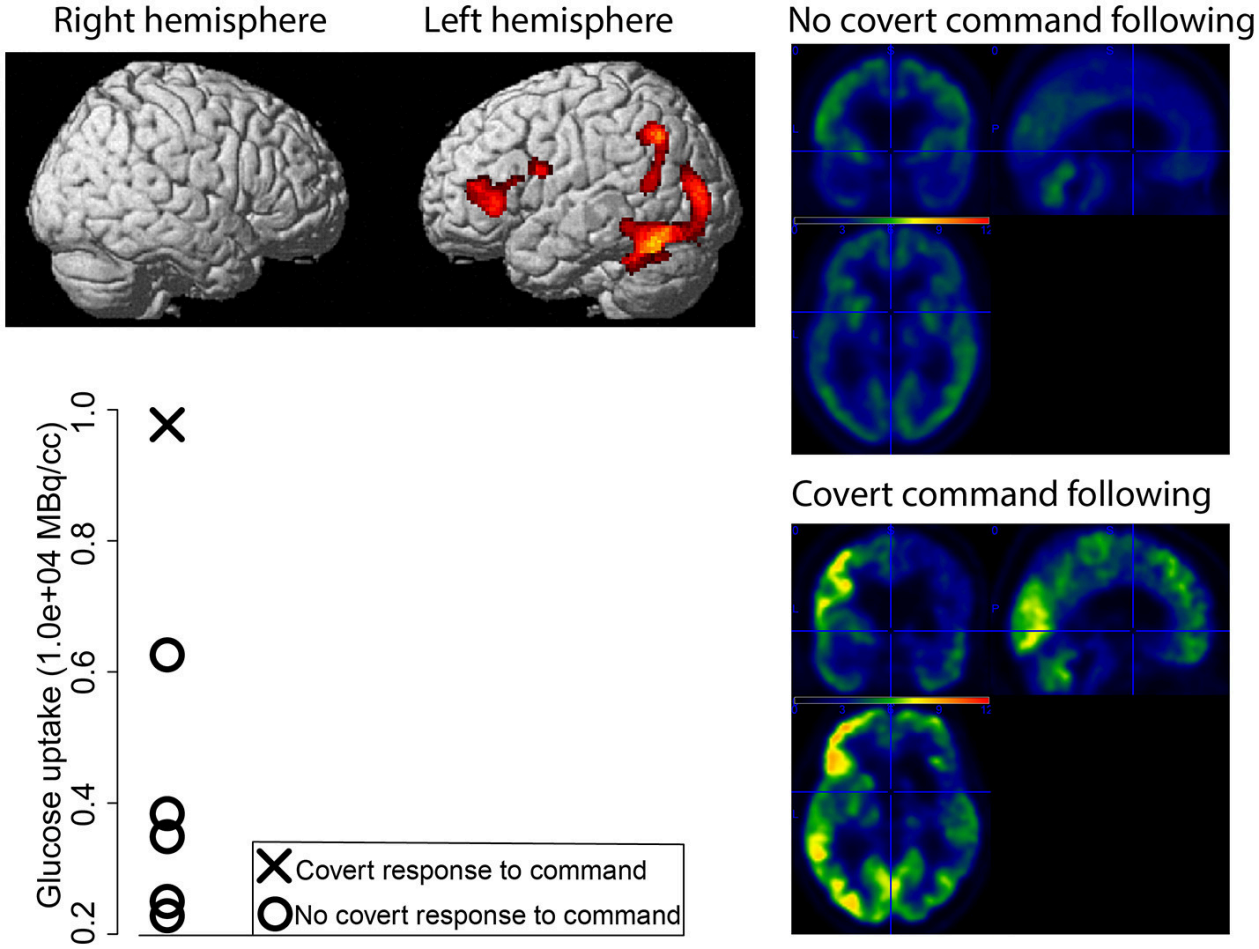
Preserved glucose metabolism (in red-yellow) as measured with FDG-PET for the MCS– patient with signs of “covert command following” compared to patients with a FDG-PET indicative of MCS without signs of “covert command following” (top left). Mean glucose uptake of the more significant cluster (in MBq/cc) for every patient (bottom left, patients with a MCS FDG-PET in absence of “covert command following” represented with circles, the MCS– patient who did show signs of “covert command following” represented with a cross). Average standardized uptake value for the patients without “covert command following” (right top), and the standardized uptake value for the patient with “covert command following” (bottom right).

**Figure 2:**
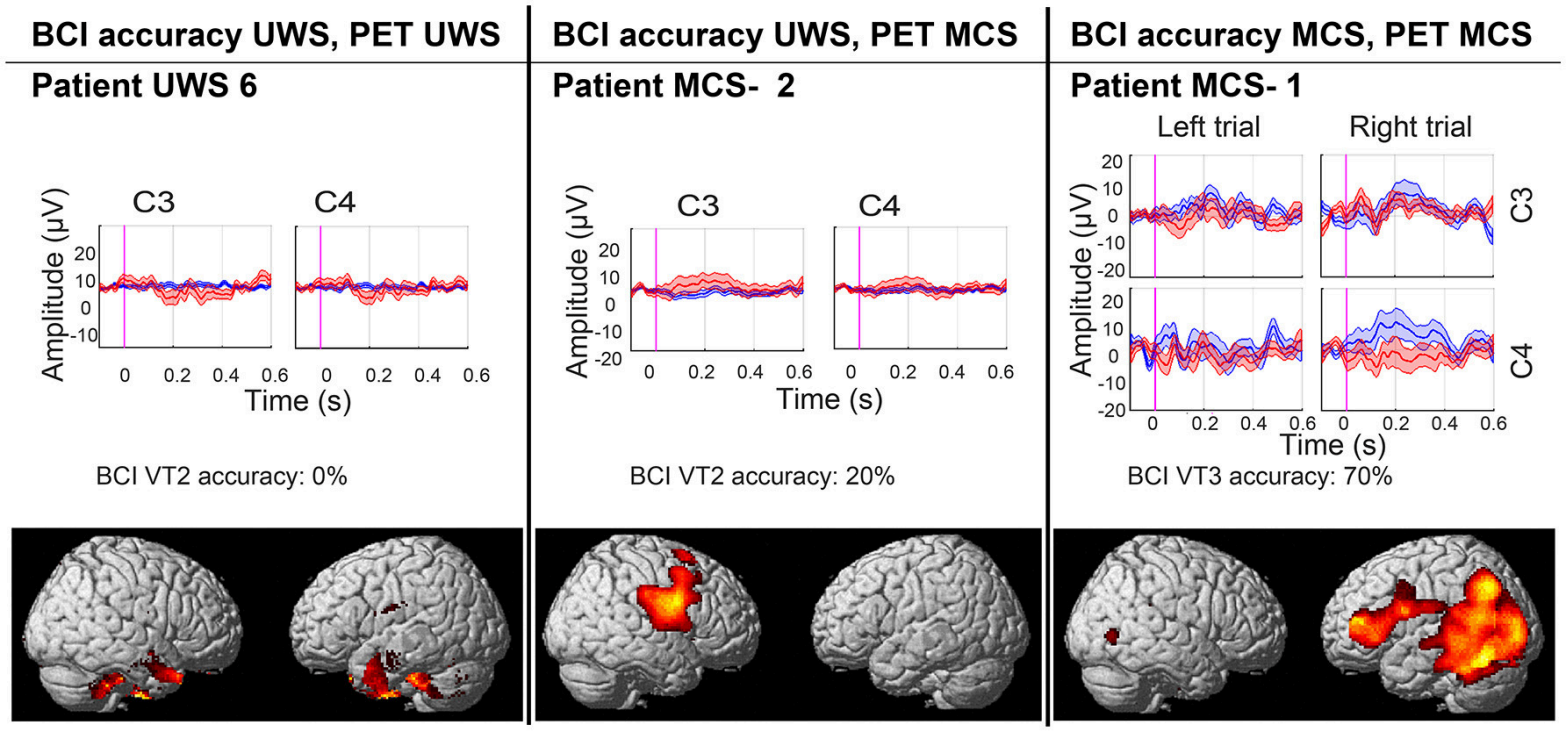
BCI performance and areas of preserved (in red-yellow) cerebral glucose metabolism compared to healthy subjects (significant at <0.001 uncorrected). Results are presented for a representative UWS (left) and MCS (middle) patient without covert response to command, and for the patient with covert response to command (right). In the ERP plot blue lines represent the P3 for the attended hand, and red line represent the P3 for the unattended hand.

Twelve patients were included in the study, of which four MCS- patients (age median = 47.5, IQR = 20 years; disease duration median = 7.5, IQR = 7.75 months; 3 males; 3 TBI, 1 anoxia), and eight UWS patients (age median = 43.5, IQR = 25.5 years; disease duration median = 50, IQR = 30.5 months; 4 males; 2 TBI, 5 anoxia, 1 hemorrhage). The VT3 was performed in only one patient (MCS1), for whom the BCI performance during the VT2 and VT3 reached 100 and 70% respectively. The BCI decoded an answer for one out of six questions, but the BCI did not decode replies during further attempts. This patient showed a preserved metabolism within the left hemisphere (i.e., language network) as compared to the other patients with a FDG-PET indicative of MCS (Figure 1). This preservation was confirmed when compared with healthy subjects (Figure 2).

All patients behaviorally diagnosed as MCS showed cortical metabolism preservation in accordance with a diagnosis of MCS. Six out of eight patients diagnosed as UWS had a FDG-PET in agreement with the CSR-R based diagnosis, while the other two patients showed preserved cortical glucose metabolism suggestive of MCS. The patients and healthy subjects used for the FDG-PET-based diagnosis did not differ in age (*Z* = 0.32, *p* = 0.75) or gender [$\chi_{\left(1\right)}^{2}$ = 1.98, *p* = 0.16]. Patients' demographics, BCI performance, and FDG-PET diagnoses are reported in Table 1. BCI responses and preserved metabolism as compared to healthy subjects are presented in Figure 2 for three patients (i.e., one UWS patient, one MCS– patient, and the patient with “covert command following”).

**Table 1:** Demographic, BCI and FDG-PET information per patient.

**ID**	**Age range**	**Disease duration**	**Etiology**	**Handedness**	**Diagnosis stability**	**VT2 [%] (# rejected trials)**	**VT3 [%] (# rejected trials)**	**FDG-PET diagnosis**
MCS– 1	40–45	60 m	TBI	Right	4/6	100 (3)	70 (1)	MCS
MCS– 2	20–25	40 m	TBI	Left	6/6	20 (1)	–	MCS
MCS– 3	55–60	8 m	Anoxia	Right	1/6	25 (42)	–	MCS
MCS– 4	55–60	70 m	TBI	?	4/6	10 (257)	–	MCS
UWS 1	65–70	3 m	Hemorrhage	Right	4/4	0 (3)	–	MCS
UWS 2	30–35	9 m	TBI	Left	5/5	20 (3)	–	MCS
UWS 3	55–60	6 m	Anoxia	?	5/5	75^+^ (0)	–	UWS
UWS 4	20–25	15 m	Anoxia	?	6/6	10 (51)	–	UWS
UWS 5	45–50	6 m	Anoxia	Right	6/6	0 (23)	–	UWS
UWS 6	65–70	5 m	Anoxia	Left	7/7	0 (21)	–	UWS
UWS 7	40–45	26 m	Anoxia	Right	6/6	40 (480^*^)	–	UWS
UWS 8	30–35	13 m	TBI	Right	6/6	10 (0)	–	UWS

*The clinical diagnosis of the patients is based on the best CRS-R of at least five assessments that were performed within the week of the BCI assessment. Fluctuations in the clinical diagnosis are presented as the proportion of best diagnosis out of the total number of assessments. Median BCI performance for the two (VT2 and VT3) paradigms and between brackets the number of rejected trials are presented together with the FDG-PET based diagnosis. Patient MCS- 1 showed signs of response to command when assessed with the BCI*.

* Very high amplitude response.

+ *artifacted by mechanical artifact*.

At the group level, the BCI performance during the VT2 paradigm was lower for UWS than for MCS patients (UWS median = 10, IQR = 30; MCS median = 22.5, IQR = 47.5; *Z* = 2.10, *p* = 0.04). When comparing the BCI performance with the FDG-PET diagnosis, the performance during the VT2 paradigm was also lower for UWS than for MCS patients (UWS median = 10, IQR = 40; MCS median = 20, IQR = 15; *Z* = 2.09, *p* = 0.04).

## Discussion

In this prospective study, we used a commercially available P3-based BCI system in a convenience sample of 12 clinically well-characterized patients with DOC. We identified a patient with signs of “covert command following,” and compared those findings to cerebral glucose metabolism preservation of patients without signs of “covert command following.”

We have found that one behaviorally MCS- patient (i.e., showing visual pursuit but no response to command at the bedside) was able to show “covert command following” using the VT3 paradigm (i.e., attended toward the left or the right stimulated hand, as requested). This patient, who showed “covert response to command,” had an FDG-PET in agreement with the diagnosis of MCS (Stender et al., 2014). This patient had already been assessed by our group about 1.5 years before the BCI assessment and had been diagnosed in a clinical state of MCS–. The week of the BCI assessment, MRI examination showed a gray matter atrophy most severe in subcortical areas and in the middle and posterior cingulum, but relatively limited in other cortical areas, suggesting a higher level of consciousness (Annen et al., 2018). The clinical EEG showed a 5 Hz rhythm, which has been associated to a higher chance of being MCS+ (as compared to MCS–; Chennu et al., 2017). The FDG-PET also showed an increase in cerebral metabolism (as compared with previous assessment), mostly pronounced in the regions of the right dorsolateral prefrontal cortex, the inferior parietal junction and the inferior temporal gyrus. These regions, suggested before to be key regions differentiating MCS– (absence of language understanding) and MCS+ (presence of language understanding) patients (Bruno et al., 2012), were also more preserved in the patient with signs of “covert command following” than in the other patients with cerebral metabolism suggestive of MCS. However, the outcome at 1 year after the BCI assessment still suggested a diagnosis of MCS–. The relatively good results of the paraclinical assessment together with the limited motor response during clinical assessment (i.e., 1/6 assessment an automatic motor reaction and 5/6 (abnormal) flexion to noxious stimulation) and severe spasticity (i.e., Modified Ashworth Scale score of 3/4 for the upper limbs and 4/4 for the lower limbs) could therefore suggest that this patient's behavior was mainly limited by her physical rather than cognitive impairments.

Previous literature have reported that about 20% of the DOC patients show covert response to command if tested using active EEG-based paradigms (Cruse et al., 2011, 2012). However, one of the main challenges in this field is the heterogeneity in data analyses and statistical assumptions used. These choices can influence the results and lead to false positives or negatives (Cruse et al., 2013; Goldfine et al., 2013), even in locked in syndrome patients assessed with the same and a different system as employed in the current manuscript (Spüler, in review). It is key to keep this in mind when interpreting such data, especially in the context of DOC patients, where such false negative or positive results might have harmful effects in the short and long term, triggering end-of-life decisions or inversely nurturing false hopes (Jox et al., 2012). One way to avoid false negatives or positives is to confront the results obtained through different techniques and/or modalities as presented here. Multimodal approaches, even if they necessitate more time and resources, may help reduce the underestimation of the patient's levels of consciousness (Stender et al., 2014; Annen et al., 2018). In the present study, the FDG-PET data ensure the validity of the presented BCI results.

The fact that only one out of twelve patients showed signs of “covert command following” [i.e., 8%, vs. 19% (Cruse et al., 2011) or vs. 30% (Spataro et al., 2018)] as previously reported in UWS patients using BCI approaches) in our small sample could be explained by the high proportion of patients with anoxic brain damage in the included sample, which previously have been reported to show “covert command following” less often than patients with a traumatic etiology (Cruse et al., 2012). When considering TBI patients only, 20% of the patients show signs of covert command following (i.e., 1 of 5 in the current study, and 2 of 10 in Cruse et al., 2011). Additionally, we included solely chronic (i.e., > 3 months after injury) DOC patients as compared to the study including acute DOC patients which suggested that 30% of the patients show “covert command following” (Spataro et al., 2018). Even if recovery of consciousness in the chronic phase of the disease can happen (Estraneo et al., 2010), recovery is more common to start in the acute phase after the injury (Whyte et al., 2013). Hence discordant results suggestive of covert command-following are expected to be more frequent in the acute phase, during which the P3 response is predictive for a good outcome (Tzovara et al., 2016). Still, the current small and heterogeneous convenience sample could limit the generalizability of the results. Especially since the provided data does not include offline analysis allowing for a tailored single-subject significance threshold for each session, the interpretation of these results remains limited. Furthermore, vigilance fluctuation (Piarulli et al., 2016) could also have an impact on the number of negative results. For behavioral assessment, it is advised to repeat the assessment at least five times, in order to avoid false negatives (Wannez et al., 2018). In this study, every patient was assessed only once with the P3 system. Moreover, the VT3 paradigm was only tested when the results for the VT2 paradigm were promising, here in one patient only. In the future, the BCI measurements should be repeated regularly to reduce false negatives as a result of arousal fluctuations, and to monitor the patient's recovery. This could aid diagnosis in the acute phase of the injury, as well as improve the quality of life of patients in the chronic phase of the disease by providing assistive technologies and communication tools (Whyte et al., 2013).

On the other hand, we would like to highlight several strong points of the current study. Both the VT2 and VT3 paradigm take only 2.4 min per session, which is much shorter than a motor imagery paradigm that usually takes about 10 min (Cruse et al., 2011, 2012), or fNIRS session which takes 9 min (Chaudhary et al., 2017). Secondly, the employed system has the potential to analyze (albeit imperfect) the data directly, and provides feedback about the patient's performance promptly. Last, the BCI results have been confronted to FDG-PET data on the single-subject level, and we have shown that neuroimaging and neurophysiological markers of consciousness and “covert command following” were in accordance with each other.

At the group level, the results for the VT2 paradigm showed higher BCI performance in MCS based on the CRS-R and/or FDG-PET than in UWS. Previous literature during various states of (un)consciousness such as sleep, anesthesia, and DOC (for review see Chennu and Bekinschtein, 2012) has shown evidence for the absence of a link between the P3 and consciousness. However, in the acute phase of the disease, outcome prediction using auditory irregularities has been successful in more than 90% of the cases (Tzovara et al., 2016). In a recent pilot study including a small sample of 12 patients, the accuracy of the vibrotactile paradigm, as employed here, was proposed to be higher in patients with an increased CRS-R score after 6 months (Spataro et al., 2018).

Together, this study highlights the interest of using a multimodal approach when interpreting results obtained through different techniques and points toward a potential added value of the VTP3 paradigm in the clinical assessment of DOC patients at the single-subject level.

## Author contributions

JA designed the work, did the acquisition, analysis, and interpretation of data for the work and drafted the work. SB and NL did a significant part of the data acquisition and revised the manuscript critically for important intellectual content. CC, MB, AT, WC, CG were involved in data analysis and revised the manuscript. SL designed the work and revised it critically for important intellectual content. All authors gave their final approval of the version to be published and agree to be accountable for all aspects of the work in ensuring that questions related to the accuracy or integrity of any part of the work are appropriately investigated and resolved.

### Conflict of interest statement

The authors declare that the hard- and software was made available by Gtec. WC is employed by g.tec Medical Engineering GmbH, CG is the CEO of g.tec Medical Engineering GmbH and g.tec Guger technologies OG. SL is on the scientific advisory board of Gtec Medical Engineering. The other authors declare that the research was conducted in the absence of any commercial or financial relationships that could be construed as a potential conflict of interest.
